# Biomolecules Responsible for the Total Antioxidant Capacity (TAC) of Human Plasma in Healthy and Cardiopathic Individuals: A Chemical Speciation Model

**DOI:** 10.3390/antiox10050656

**Published:** 2021-04-23

**Authors:** Enrico Prenesti, Silvia Berto, Fabio Gosmaro, Marco Bagnati, Giorgio Bellomo

**Affiliations:** 1 Department of Chemistry, University of Turin, Via Pietro Giuria 5, 10125 Turin, Italy; enrico.prenesti@unito.it (E.P.); fabiogos@yahoo.it (F.G.); 2 Istituto Professionale di Stato per Servizi Alberghieri e Ristorazione G. Giolitti, Piazza IV Novembre, 12080 Mondovì, Italy; 3 Clinical Chemical Analysis Laboratory of the University Hospital “Maggiore della Carità”, University of Eastern Piedmont Amedeo Avogadro, C.so Giuseppe Mazzini, 18, 28100 Novara, Italy; marco.bagnati@maggioreosp.novara.it (M.B.); bellomo.giorgio@gmail.com (G.B.)

**Keywords:** total antioxidant capacity, antioxidant biomolecules, CUPRAC, human plasma, healthy individuals, chemical speciation model, cardiovascular surgery, vitamins

## Abstract

(1) Background: Much effort has been expended to investigate the antioxidant capacity of human plasma, attempting to clarify the roles of both metabolic and food substances in determining defenses against oxidative stress. The relationship between the total antioxidant capacity (TAC) and the concentrations of redox-active biomolecules in the human plasma of healthy and cardiopathic individuals was investigated in the present study to develop a chemical speciation model. (2) Methods: Plasma was collected from 85 blood donors and from 25 cardiovascular surgery patients. The TAC was measured using the CUPRAC-BCS (CUPric Reducing Antioxidant Capacity — Bathocuproinedisulfonic acid) method. Biomolecule concentrations were determined via visible spectrophotometry or HPLC/RP techniques. The relationship between the TAC and the concentrations was defined by applying a multiple regression analysis. The significance of the variables was first tested, and chemical models were proposed for the two datasets. The model equation is $\mathrm{TAC}=\sum_{i}\beta_{i}\cdot \left[A_{i}\right]$, where β_i_ and [A_i_] are the electronic exchange and the molar concentrations of the i^th^ antioxidant component, respectively. (3) Results: The major contributions to the TAC, ~80%, come from endogenous compounds in both healthy and cardiopathic individuals, whereas the contributions from exogenous compounds were different between the two datasets. In particular, γ-tocopherol showed a different role in the chemical models developed for the two groups.

## 1. Introduction

As reported by recent reviews [1,2], much effort has been expended by scientists to investigate the antioxidant capacity of human plasma in the past two decades, particularly in attempts to clarify the roles of both metabolic and food substances in determining defenses against oxidative stress. Oxidative stress is an undesirable condition caused by an imbalance between free radicals, commonly produced by aerobic metabolism, and scavenger biomolecules. Much speculation on the clinical meaning and utility of Total Antioxidant Capacity (TAC) appears in the specialized literature and was previously widely discussed by the authors ([3] and references therein). TAC is conceived as a method-dependent quantity that provides information on the redox status of a fluid according to a simple measurement principles associated with a particular measuring apparatus. TAC has been measured in food, beverages, and body fluids according to various test protocols. The ultimate common goal is to collect information to prevent diseases, promote health, and increase longevity. Some papers [4,5,6] have shown an inverse correlation between dietary TAC and various type of cancer incidence; nevertheless, it seems risky to draw generalized conclusions by assuming the TAC of food can be used as a prevention tool of certain diseases.

Our research group decided to apply its long-time expertise in chemical speciation modeling [7,8,9] to split the TAC of human plasma into contributions ascribable to each physiological redox-active substance. The developed speciation model also allows the contributions to the TAC of different metabolic and food substances to be distinguished. Speciation of the TAC quantity was performed for healthy individuals (used as reference [10]). Plasma from cardiovascular surgery patients was also used to obtain a dataset different from that gathered from healthy individuals in order to increase knowledge about the applicability of the chemical model developed. The TAC was measured using the CUPRAC-BCS (CUPric Reducing Antioxidant Capacity—Bathocuproinedisulfonic acid) method, which has been previously optimized, validated, and tested [11,12]. As stated by Apak et al. [13], the CUPRAC method is recognized as excellent in terms of its pH, which is close to a physiological one; favorable redox potential; accessibility and stability of reagents; and applicability to lipophilic antioxidants as well as hydrophilic ones. 

Operationally, plasma from 85 blood donors (47 males and 38 females) was collected. The redox-active biomolecules considered in the development of the chemical model were uric acid, total bilirubin, thiols (thiol group containing molecules expressed as L-glutathione), retinol (vitamin A), α-tocopherol (vitamin E), γ-tocopherol (vitamin E), lycopene (a carotenoid), β-carotene (pro-vitamin A), and L-ascorbic acid (vitamin C). Their concentrations were determined via routine visible spectrophotometry or HPLC/RP techniques. 

The chemical speciation model was built using the multiple regression analysis technique. The concentrations of the antioxidants were weighted for the corresponding electronic exchange related to the test reaction and the TAC was then modeled using the equation $\mathrm{TAC}=\sum_{i}\beta_{i}\cdot \left[A_{i}\right]$, where β_i_ and [A_i_] are the redox factors (the electronic exchange values, henceforth RF_i_) and the molar concentrations of the i^th^ antioxidant component, respectively. The significance of antioxidants as predictors of TAC was previously tested by way of a preliminary multiple regression analysis.

Furthermore, plasma samples from 25 cardiovascular surgery patients (17 males and 8 females) were collected and subjected to the same measurements, data treatments, and modeling as those of the healthy individuals. The group of cardiovascular surgery patients was selected on the assumption that this condition probably implied a detectable imbalance in the oxidative state of plasma. The means of the TAC values of the cardiovascular surgery patients stood at high values. This fact may hint at a redox imbalance due to different involvement of plasma antioxidants in heart disease (which necessitated surgery in these patients) and suggests new areas of investigation. The main aim of the work was to enhance the information on the extracellular redox buffering capacity of the human plasma. The developed speciation model allowed (1) identification of the biomolecules responsible of the redox buffer capacity of human plasma, (2) identification of the weight of each redox-active biomolecule in determining different buffer effect, (3) distinguishing the TAC between food and metabolic sources.

## 2. Materials and Methods

### 2.1. Chemicals

Cu(II) sulphate pentahydrate (purity ≥ 98%), bathocuproine disulfonic acid (BCS, purity ≥ 98%), PBS (phosphate-buffered saline 0.1 mol L^−1^), L-ascorbic acid (purity ≥ 98%), bilirubin (purity ≥ 96%), uric acid sodium salt (purity ≥ 98%), human serum albumin (HSA, purity ≥ 99%), retinol (synthetic, purity ≥ 95%, for HPLC), lycopene (purity ≥ 90%, for HPLC), (±)-α-tocopherol (synthetic, purity ≥ 96%, for HPLC), (+)-γ-tocopherol (synthetic, purity ≥ 96%, for HPLC), (±)-6-hydroxy-2,5,7,8-tetramethylchromane-2-carboxylic acid (Trolox, purity ≥ 97%), β-carotene (synthetic, purity ≥ 96%), meta-phosphoric acid (purity ≥ 99%, lumps), ortho-phosphoric acid (purity ≥ 85%), potassium dihydrogen phosphate (purity ≥ 99%), ethylenediaminetetraacetic acid (purity ≥ 98%), 5,5′-dithiobis(2-nitrobenzoic acid) (DTNB, purity ≥ 99%), L-glutathione reduced (purity ≥ 98%), and glacial acetic acid were purchased from Sigma-Aldrich (St. Louis, MO, USA).

Acetonitrile, dichloromethane, methanol, n-hexane, and ethanol (solvents for analytical liquid chromatography), Cu(II) reference solution (1000 ± 1) mg L^−1^, and pH-metric buffer solutions (pH 4.01 and 9.00 at 20 °C) were supplied by Merck (Darmstadt, Germany). 

ADVIA^®^ Chemistry Uric Acid Concentrated Reagent and ADVIA Chemistry Total Bilirubin_2 were purchased from Siemens Healthcare Diagnostics Inc. (Tarrytown, NY, USA).

Solutions were prepared in grade A glassware and diluted with ultrapure water (MilliQ quality). 

Details for the preparation of the solutions used in the TAC determination are fully described in Reference [11]. 

### 2.2. Equipment

A clinical chemistry auto-analyzer ADVIA 2400 Chemistry System, provided by Siemens (Munich, Germany), with 2400 test/h capacity and two reagent trays, was used in the present study. 

Visible photometric determinations of thiols and of the redox factors involved in the speciation of the TAC were carried out using a V-550 spectrophotometer provided by Jasco (Cremella, LC, Italy), with a cuvette optical path length of 10 mm.

The determinations of both lipid-soluble antioxidants and L-ascorbic acid were carried out using the Hewlett-Packard 1100 UV HPLC System, provided by Agilent (Santa Clara, CA, USA), including the HP G1322A vacuum degasser, HP G1310A isocratic pump, HP G1313A autosampler, HP G1326A thermo-stated column compartment, and HP G1314A UV-VIS detector.

A pH meter pH-211 Hanna Instruments (Ronchi di Villafranca Padovana, PD, Italy) equipped with a Porotrode pH glass electrode by Metrohm (Origgio, VA, Italy) was also used.

### 2.3. Procedures

#### 2.3.1. Determination of Uric Acid, Total Bilirubin, and TAC.

The visible photometric determinations of uric acid, total bilirubin, and TAC were carried out using ADVIA 2400. The procedures used for the TAC, uric acid, and bilirubin quantifications are fully described in References [3,11,12]. 

#### 2.3.2. Determination of Lipophilic Antioxidants

The quantifications of lipophilic antioxidants, namely retinol, γ-tocopherol, α-tocopherol, lycopene, and β-carotene, were conducted according to the method described by Ortega et al. [14].

Stock solutions were prepared of retinol 1.0 mmol L^−1^ in dichloromethane, γ-tocopherol 2.0 mmol L^−1^ and α-tocopherol 4.0 mmol L^−1^ in ethanol, and lycopene 0.05 mmol L^−1^ and β-carotene 0.1 mmol L^−1^ in dichloromethane. The stock solutions were further diluted with acetonitrile:dichloromethane:methanol (67:19:14 *v*:*v*:*v*) (henceforth ADM mixture). Final concentrations (µmol L^−1^) in the flask were 3.00 for retinol and γ-tocopherol, 40.0 for α-tocopherol, and 1.00 for lycopene and β-carotene.

Immediately after blood collection and centrifugation, 200 µL of plasma was added to 300 µL of MilliQ water and 500 µL of ethanol and vortexed for 10 s. Following this step, 2000 µL of hexane was added and the extraction of lipid-soluble antioxidants was completed through vortexing for 2 min and centrifugation at 3000 rpm for 1 min. After extraction, 1000 µL of supernatant was withdrawn and evaporated under nitrogen flow. Finally, 100 µL of ADM mixture was used to bring the extract into solution. The extract samples were directly analyzed or kept at −80 °C until analysis.

Lipid-soluble antioxidant contents were determined using a reversed-phase column (ECLIPSE XDB—C18 4.6 × 100 mm, 3.5 µm, by Agilent, SC, USA) at 27 °C. The mobile phase was the ADM mixture with glacial acetic acid 1 g L^−1^ at a flow rate of 1.8 mL min^−1^. Working wavelengths were, respectively, 326 nm for retinol, 292 nm for γ-tocopherol and α-tocopherol, and 460 nm for lycopene and β-carotene. Sample injection volume was 20 µL and the run time was 8 min.

#### 2.3.3. Determination of L-Ascorbic Acid

The determination of L-ascorbic acid was conducted as described by Iwase et al. [15].

A stock solution 10 mmol L^−1^ L of ascorbic acid was prepared in water. The stock solution was further diluted with water up to 40 µmol L^−1^.

Immediately after blood collection and centrifugation, 200 µL of plasma were deproteinized with 400 µL of meta-phosphoric acid (10% wt/wt), vortexed for 15 s, and centrifuged at 10,800 rpm for 7 min. Following this step, 350 µL of supernatant were directly analyzed or kept in vials at −80 °C until analysis.

L-ascorbic acid content was determined using a reversed-phase column (Zorbax SB C18 Stable Bond 4.6 × 250 mm, 5 µm; Agilent, Santa Clara, USA) at 27 °C. KH_2_PO_4_ 20 mmol L^−1^ and 0.2 mmol L^−1^ of EDTA solution (pH = 3.0 adjusted with ortho-phosphoric acid solution) was used as the mobile phase. The flow rate was set at 1 mL min^−1^ and the wavelength used for the photometric detection was 244 nm. Sample injection volume was 20 µL and the run time was 14 min.

#### 2.3.4. Determination of Thiols

The determination of thiol groups was made according to the method described by Eyer et al. [16] based on Ellman’s method. DTNB (5,5′-dithiobis(2-nitrobenzoic acid); Ellman’s reagent) solution at 2.5 mmol L^−1^ in PBS 10 mmol L^−1^ (pH 7.4) was freshly prepared and kept in the dark and on ice. Pretreatment of plasma was not required. For each unknown sample to be tested, a tube containing 2000 μL Ellman’s reagent solution and 2000 μL PBS 10 mmol L^−1^ (pH 7.4) was prepared. Following this step, 100 μL of each unknown sample was added to the separate test tubes containing Ellman’s reagent solution. As a blank, 100 μL of unknown sample was added to 2000 μL of PBS 10 mmol L^−1^ (pH 7.4). After incubation at room temperature and in the dark for 5 min, absorbance at 420 nm was measured. Finally, the concentration of thiols in the sample was calculated using the molar absorption coefficient of 5-thio(2-nitrobenzoic acid) (14,150 mol^−1^ L cm^−1^) formed by the reaction of DTNB with analytes. Calibration was done using reduced L-glutathione as a standard molecule (thiol content is expressed in L-glutathione equivalent units).

#### 2.3.5. Determination of Redox Factors

The redox reaction between Cu(II) and the biomolecules affecting the TAC can be expressed by the general Equation (1):

a Cu(II)-BCS + b S_rid_ → c Cu(I)-BCS + d S_ox_ (1)

where Cu(II)/(I)-BCS are the copper complexes with BCS; S_rid/ox_ indicates the redox-active biomolecules in the sample; and a, b, c, and d are the redox factors involved in the reaction. A solution of Cu(II)-bathocuproine was treated with a known amount of reducing molecule. The variation of the absorbance at 478 nm was measured for 4 min according to the CUPRAC-BCS method. The actual amount of Cu(I) produced was then obtained using the molar absorption coefficient of Cu(I)-bathocuproine [12] and plotted against the concentration of the reacting molecule. The slope of the experimental curve obtained corresponds to the number of electrons exchanged during the redox reaction between the reducing molecule under study and the Cu(II) cation. In particular, the redox factors were estimated for uric acid, bilirubin, human serum albumin (HSA-SH, as thiol groups containing molecule), α-tocopherol, γ-tocopherol, and L-ascorbic acid.

The studies of the redox reactions were carried out using six standard solutions of each reducing molecules, prepared and analyzed in three replicates at the following concentrations: (1) uric acid sodium salt 0.2, 0.5, 1.0, 1.5, and 2.0 mmol L^−1^; (2) bilirubin 2.5, 5.0, 12.5, 25, 50, and 100 µmol L^−1^; (3) α- or γ-tocopherol 0.2, 0.4, 0.6, 0.8, 1.0, and 1.3 mmol L^−1^, dissolved in dichloromethane; (4) HSA-SH 0.3, 0.6, 0.9, 1.2, 1.5, and 1.8 mmol L^−1^; and (5) L-ascorbic acid 0.1, 0.2, 0.3, 0.5, 0.7, and 1.0 mmol L^−1^. 

In order to evaluate the possible effect of the solvent, a separate experiment was conducted on (±)-6-hydroxy-2,5,7,8-tetramethylchromane-2-carboxylic acid (Trolox, the water-soluble derivative of α-tocopherol). The electronic exchange was shown to be unaffected by the presence of dichloromethane.

### 2.4. Subjects

According to the International Federation of Clinical Chemistry and Laboratory Medicine (IFCC), the a priori approach [17] was used for the selection of reference individuals. In this study, the blood donor population was chosen to represent the healthy adult population, as previously reported [3]. Plasma from 25 cardiovascular surgery patients (17 males and 8 females) was also collected.

The present study used leftover plasma samples accompanied by anonymized data. Plasma samples were always collected during routine sessions scheduled for purposes unrelated to the study. The collection of experimental data was carried out to increase knowledge of the redox chemistry of plasma, without diagnostic or therapeutic goals.

### 2.5. Plasma Sample Collection and Preparation

Blood from apparently healthy (blood donors) and cardiopathic subjects was gathered into lithium–heparin-containing tubes. The blood was centrifuged at 3500 rpm for 6 min at 15 °C, and plasma was obtained as a supernatant.

### 2.6. Statistical Data Treatment 

The datasets of both healthy and cardiopathic individuals were subjected to a statistical treatment to investigate the relationship between the TAC quantity and the concentrations of the redox-active biomolecules. This check was conducted by way of multiple regression analysis. The linear model used was forced through the origin and the model building was set in the weighted regression modality. The backward removal procedure (removal criterion: *p* ≥ 0.10) was adopted to select the significant predictors.

### 2.7. Software 

Origin 6.1. (by OriginLab Corporation, Northampton, MA, USA) and SPSS Statistics 17.0 (by SPSS, Segrate, MI, Italy) were used for data processing and presentation. 

## 3. Results

### 3.1. Analytical Results

TAC, uric acid, total bilirubin, thiol groups (HSA-SH), retinol, γ-tocopherol, α-tocopherol, lycopene, β-carotene, and L-ascorbic acid were quantified, as described before, in the two categories of individuals: (1) blood donors (healthy individuals), 85 plasma samples; (2) cardiovascular surgery patients (cardiopathic individuals), 25 plasma samples. Table 1 and Table 2 show the descriptive statistics of the two datasets.

### 3.2. Significance of Antioxidants as Predictors of Healthy Individuals’ TAC

The dataset of healthy individuals was subjected to a statistical treatment to investigate the relationship between the TAC quantity and the concentration of antioxidants. This investigation was preliminary to the assessment for the speciation model (optimized by way of a hard-modeling procedure) and aimed to identify those molecules that significantly contributed to the TAC definition. 

A multiple regression analysis was applied with the aim of identifying the relationship between the TAC quantity (dependent variable) and the concentrations of nine predictors (independent variables): urate, total bilirubin, thiol groups, retinol, γ-tocopherol, α-tocopherol, lycopene, β-carotene, and L-ascorbic acid. Concentrations of the predictors were combined assuming a linear model, as follows:

[TAC] = β_1_[uric acid] + β_2_[total bilirubin] + β_3_[thiols] + β_4_[retinol] + β_5_[γ-tocopherol] + β_6_[α-tocopherol] + β_7_[lycopene] + β_8_[β-carotene] + β_9_[L-ascorbic acid] (2)

where β_i_ are the regression parameters optimized by the multiple regression analysis. In particular, the model building was set in the weighted regression modality, forced through the origin, and used a backward removal procedure (removal criterion: *p* ≥ 0.10). 

Table 3 and Tables S1–S3 in the Supplementary Materials show the results of the multiple regression analysis obtained using the healthy individuals’ dataset. Model 5 described the chemical system under study well. The molecular predictors significantly contributing to the TAC were uric acid, total bilirubin, thiol groups, α-tocopherol, and L-ascorbic acid.

### 3.3. Determination of the Redox Factors

The redox reaction between Cu(II) and urate ions was previously studied and the results are reported in Reference [12]. The redox factors estimated for the other biomolecules are collected in Table 4.

The electronic exchange extent evaluated at 4 min, that is the time reaction of the CUPRAC-BCS method, is not always represented by a whole number. It can be a fractional number because not all the redox reactions achieve completion in 4 min, as observed for bilirubin and thiols (see the Supplementary Materials file, Tables S4 and S5). Within the thiols, the single cysteine residue of HSA-SH is the most abundant plasma thiol, but this group is partially unavailable for the redox reaction with Cu(II) cation [18].

### 3.4. Speciation Model of Healthy Individuals’ TAC

The correlation between the TAC and the concentration of those redox-active biomolecules found to be significant predictors of the TAC was modeled *via* multiple regression analysis according to the following equation:

(3) $$\mathrm{TAC}=\underset{i}{\sum }{\mathrm{RF}}_{i}\cdot \left[A_{i}\right]$$

where RF_i_ and [A_i_] are the redox factors and the molar concentrations of the i^th^ antioxidant component, respectively. As to the modeling conducted using Equation (3), in which the coefficients are unrelated to the chemistry of the system (soft model, based on a numerical optimization), the constraint imposed using of the redox factors as parameters ensured control and chemical consistency (hard model, responsive to the phenomenal reality). According to the hard modeling, the equation for the multiple regression analysis was (statistical details of the model are reported in Table 5):

[TAC] = 2.0[uric acid] + 7.4[total bilirubin] + 0.6[thiols] + 2.0[α-tocopherol] + 2.0[L-ascorbic acid](4)

The relative error percentage (henceforth RE%) between each measured value and predicted value was calculated as follows:

(5) $$\mathrm{RE}\%=\frac{x_{m}-x_{p}}{x_{m}}\times 100$$

where x_m_ is the measured value and x_p_ is the predicted value. The mean of relative error percentage (MRE%=RE%/n, n = number of measures) of the model was found to be equal to −1.3%.

**Figure 1:**
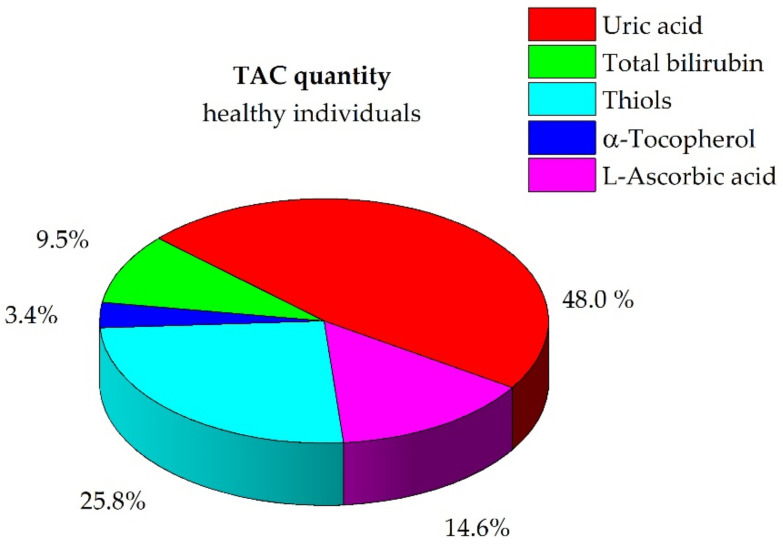
Pie chart for the speciation of TAC in healthy individuals. Percentage expressed as mean (details in Table S6).

The contributions of the redox-active biomolecules to the TAC of plasma were estimated using the hard speciation model. Figure 1 and Table S6 show the TAC in terms of each molecule under study expressed as a percentage and show how the major contributions to the TAC come from endogenous compounds, namely uric acid, total bilirubin, and thiol groups containing molecules (approximately 83% of the TAC). Contributions from exogenous compounds, α-tocopherol and L-ascorbic acid, played a role of approximately 17% only.

### 3.5. Significance of Antioxidants as Predictors of Cardiopathic Individuals’ TAC

The dataset of cardiopathic individuals was subjected to the same statistical treatment as the dataset of healthy subjects. Table 6 and Tables S7–S9 show the results of the multiple regression analysis conducted on the dataset. Model 5 describes the chemical system under study well, and the molecular predictors significantly contributing to the TAC were uric acid, total bilirubin, thiol groups, γ-tocopherol, and L-ascorbic acid. The output of the multiple regression analysis excluded α-tocopherol as significant predictor, but included γ-tocopherol.

### 3.6. Speciation Model of Cardiopathic Individuals’ TAC

The correlation between the TAC and the concentration of the redox-active biomolecules was modeled via multiple regression analysis according to Equation (3). The model equation for the group of cardiovascular surgery patients was as follows (statistical details of the model are reported in Table 7):

[TAC] = 2.0[uric acid] + 7.4[total bilirubin] + 0.6[thiols] + 2.0[γ-tocopherol] + 2.0[L-ascorbic acid](6)

The mean of relative error percentage (MRE%, see Equation (5)) of the model was found to be equal to −38.6%, suggesting a significant different from the outcome obtained using the data of the healthy subjects. 

**Figure 2:**
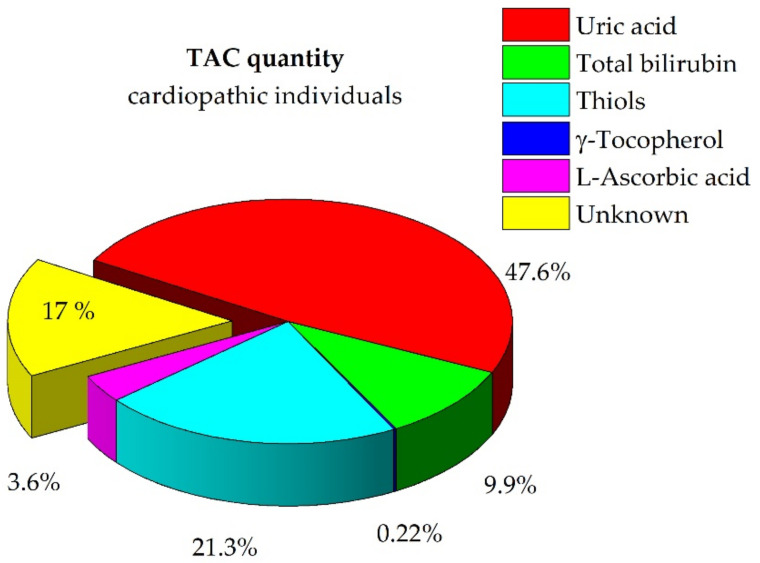
Pie chart for the speciation of the TAC quantity in cardiovascular surgery patients. Percentage expressed as mean (details in Table S10).

The quantitative contributions of each redox-active biomolecule to the TAC of plasma were estimated. Figure 2 and Table S10 show the TAC in terms of contribution of each biomolecule expressed as a percentage. The major contributions to the TAC, approximately 79%, were from endogenous compounds, namely uric acid, total bilirubin, and thiol groups from HSA-SH, as previously observed for healthy individuals. Contributions from exogenous compounds, γ-tocopherol and L-ascorbic acid, played a role of approximately 4% only. As previously argued [3], this finding confirms the relevance of these endogenous compounds in maintaining the buffered redox status of plasma, while the role of the food compounds was found to be slight. The chemical model optimized for cardiopathic subjects failed to explain approximately 17% of the TAC.

## 4. Discussion

A chemical speciation model was developed to explain the TAC in term of contributions of single redox-active biomolecules, for both healthy and cardiopathic subjects. The descriptors differ for the two groups examined, promptly indicating distinctive features. Table 8 reports mean values (males’ and females’ data mixed) of the TAC and of the redox-active biomolecules’ concentrations, to highlight the discrepancies observed between the healthy and cardiopathic subjects.

The mean TAC value for the cardiovascular surgery patients group was higher than that of the healthy subjects. The relative difference percentage RD% was 18.9 (last column of Table 8). This likely reveals a situation of metabolic imbalance caused by both the pathology and the surgery stress [3]. As regards endogenous molecules, a rough inspection of Table 8 reveals different trends: uric acid and total bilirubin showed an increase of about 12%, while HSA-SH (thiols) showed the opposite behavior with a RD% of −25.5. It is likely that uric acid and total bilirubin concentrations increase to restore the plasma antioxidant capacity impacted by the pathology. In contrast, the lowering of plasma HSA-SH concentration might be correlated with albuminuria, as reported in a study by Jackson et al. [19]. An increased excretion of HSA-SH in urine might in fact be a marker of cardiopathic conditions related to heart failure. The lowering of HSA-SH levels is probably counterbalanced by the increase of uric acid and total bilirubin to maintain plasma homeostatic redox conditions.

Appreciable differences in the distribution of food molecules (nutrients and non-nutrients) can also be identified, and two different trends can be observed in this group of exogenous biomolecules. The biggest difference was recorded for L-ascorbic acid, but other species, namely lycopene and β-carotene, also showed remarkable lowering in their plasma concentrations. These results are coherent with the food restrictions to which surgery patients are subjected. A countertendency was observed for molecular components related to vitamin E: α-tocopherol decreased (RD% = −61.7) while γ-tocopherol increased (RD% = 39.1). It is of note that α-tocopherol is the most active chemical form of vitamin E, and it is not surprising that it is consumed under exceptional oxidative stress conditions. Variation of both α- and γ-tocopherol concentrations can be associated with cardiovascular risk, as stated in the literature [20]. The metabolisms of α- and γ-tocopherol are not yet completely clarified, even if a specific role of γ-tocopherol seems to be emerging. Our findings support the abovementioned hypothesis. It is likely that the increase of the γ-tocopherol concentration in plasma could be linked to the protective role of this molecule for the circulatory system. In this case, the organism would dispose of a defensive reactant capacity to tackle the disease rather than the stress related to the surgery. In support of this hypothesis, Liu et al. found that mixed tocopherols were more potent in preventing platelet aggregation than α-tocopherol alone [21], thus underlining the efficacy of a natural tocopherol blend (including γ-tocopherol) in maintaining cardiovascular trophism. Furthermore, γ-tocopherol and its major metabolites can also reduce inflammation by inhibiting the cyclooxygenase-2 enzyme, which is central to the inflammatory processes associated with vascular disease. The ability of γ-tocopherol to moderate the production of this inflammatory mediator is not shared by α-tocopherol [22], and this could have implications.

The chemical model developed for the cardiovascular surgery patients was not fully satisfying, as expected, since it failed to explain 17.3% of TAC. Moreover, the mean of the relative error percentage (MRE%) was equal to −38.6%. This value differed significantly from that obtained using the blood donors’ dataset. The MRE% obtained via hard modeling of cardiovascular surgery patient dataset was remarkably high, suggesting a systematic error in the procedure, likely linked to unidentified predictors playing a significant role in the chemical speciation model, as discussed below.

## 5. Conclusions

The developed chemical model allowed us to draw some conclusions. The major contributions to the TAC come from endogenous compounds—uric acid, total bilirubin, and thiol groups (HSA-SH)—contributing approximately 80% for both datasets. In healthy subjects, the contribution from exogenous compounds—α-tocopherol (the most bioactive component of vitamin E group) and L-ascorbic acid (vitamin C)—was only about 18%. The results showed a sharp imbalance in favor of metabolic species, indicating a minor role of food substances in the redox buffering action of human plasma. This is not surprising when one considers that the human plasma is an extracellular buffered fluid that collects large amounts of redox-active waste substances derived from catabolism. For the sake of clarity, it is worth bearing in mind that many nutrients are precursors of metabolic compounds and serve metabolic roles different than those considered in this work.

The redox factors expressly evaluated for the reactions involved in the TAC definition anchor the model to chemical phenomena, and this constraint allows outcomes to be linked to a biomedical meaning. The lack of explanation of the TAC of cardiovascular surgery subjects, in term of biomolecules, is not surprising. Considering their pathologies, surgery, and hospitalization, this kind of patients were probably subjected to food restrictions, reduced mobility, and pharmacological therapy. The drugs involved in specific therapy for cardiovascular surgery patients (such as coumarins, heparin, salicylates, nitrosalicylates, diuretics, ACE inhibitors, calcium antagonists, β-blockers, nitroglycerin, and so on), the metabolic disorder implying overproduction of free radicals, and the anomalous nutrient intake poor in natural antioxidants will all affect the TAC value and its speciation. The group of cardiovascular surgery individuals was chosen based on their condition probably involving a significant imbalance of the oxidative status of plasma, with the main aim of testing the reliability of the speciation model under development.

TAC is a parameter of redox reactivity that is simple to measure and suitable for routine use. Even though TAC is a method-dependent quantity, we believe its usefulness in clinical chemistry can be maximized through the development of a comprehensive chemical model. The present investigation has improved the knowledge of the relationship between TAC value and the concentration of plasma redox-active biomolecules able to reduce Cu(II) to Cu(I) under the set conditions of the CUPRAC-BCS method. This chemical speciation widens the scope of knowledge and provides biochemists and physicians further information about the possible role of plasma redox-active biomolecules. Homeostasis can be considered from a chemical perspective, since an organism aims to maintain a beneficial chemical equilibrium among substances needed to perform specific biological functions. In this way, illness can be regarded as an alteration of a biochemical equilibrium, and an altered distribution of biomolecules is simply a sign of a defensive, although ineffective, response to an anomalous and damaging event. 

The approach set up here can be applied in the future to other pathologies, and thus strengthened in its analytical and forecasting capabilities.

## Figures and Tables

**Table 1:** Descriptive statistic of the dataset obtained from 85 blood donors (males’ and females’ data mixed, healthy individuals).

Quantity	Median	Mean	Std. Dev. ^1^	Min	Max	n
TAC (µmol L^−1^)	974	1002	164	717	1379	85
Uric acid (µmol L^−1^)	258	266	70	142	447	85
Total bilirubin (µmol L^−1^)	12	14	9	5	82	85
Thiols (µmol L^−1^)	463	462	63	302	596	85
Retinol (nmol L^−1^)	1834	1832	443	813	3168	85
γ-Tocopherol (nmol L^−1^)	530	775	971	10	5799	85
α-Tocopherol (nmol L^−1^)	17,934	18,048	3510	10,271	28,126	85
Lycopene (nmol L^−1^)	1731	1965	840	630	4479	85
β-Carotene (nmol L^−1^)	279	336	196	61	916	85
L-Ascorbic acid (µmol L^−1^)	66	76	49	11	245	85

^1^ Std. dev. = standard deviation.

**Table 2:** Descriptive statistic of the dataset obtained from 25 cardiovascular surgery patients (males’ and females’ data mixed, cardiopathic individuals).

Quantity	Median	Mean	Std. Dev. ^1^	Min	Max	n
TAC (µmol L^−1^)	1194	1235	451	576	1981	25
Uric acid (µmol L^−1^)	331	303	139	89	558	25
Total bilirubin (µmol L^−1^)	12	16	12	3	60	25
Thiols (µmol L^−1^)	337	368	88	230	597	25
Retinol (nmol L^−1^)	1037	1202	586	222	2362	25
γ-Tocopherol (nmol L^−1^)	867	1272	1417	10	5497	25
α-Tocopherol (nmol L^−1^)	10,431	11,160	3376	5728	17,974	25
Lycopene (nmol L^−1^)	530	554	302	48	1241	25
β-Carotene (nmol L^−1^)	81	130	134	22	638	25
L-Ascorbic acid (µmol L^−1^)	16	20	11	8	46	25

^1^ Std. dev. = standard deviation.

**Table 3:** Regression parameters optimized by the multiple regression analysis for healthy individuals.

Model ^1^		Unstandardized Coefficients	Standard Deviation Error	Standardized Coefficients	*t* Value	Significance
1	Uric acid	2.668	0.086	0.653	31.087	0.000
	Total bilirubin	4.469	0.615	0.067	7.267	0.000
	Thiols	0.325	0.069	0.139	4.688	0.000
	Retinol	12.737	13.353	0.022	0.954	0.343
	γ-Tocopherol	−2.916	5.564	−0.003	−0.524	0.602
	α-Tocopherol	5.203	1.753	0.088	2.968	0.004
	Lycopene	−3.162	7.574	−0.006	−0.417	0.678
	β-Carotene	40.103	30.601	0.015	1.311	0.194
	L-Ascorbic acid	0.642	0.122	0.055	5.264	0.000
2	Uric acid	2.668	0.085	0.653	31.255	0.000
	Total bilirubin	4.487	0.610	0.068	7.355	0.000
	Thiols	0.318	0.067	0.137	4.733	0.000
	Retinol	12.808	13.280	0.022	0.964	0.338
	γ-Tocopherol	−2.768	5.523	−0.003	−0.501	0.618
	α-Tocopherol	4.966	1.650	0.084	3.010	0.004
	β-Carotene	38.268	30.121	0.014	1.270	0.208
	L-Ascorbic acid	0.656	0.117	0.056	5.612	0.000
3	Uric acid	2.671	0.085	0.654	31.522	0.000
	Total bilirubin	4.492	0.607	0.068	7.400	0.000
	Thiols	0.313	0.066	0.134	4.735	0.000
	Retinol	11.907	13.095	0.021	0.909	0.366
	α-Tocopherol	5.043	1.635	0.085	3.085	0.003
	β-Carotene	35.146	29.328	0.013	1.198	0.234
	L-Ascorbic acid	0.665	0.115	0.057	5.770	0.000
4	Uric acid	2.689	0.082	0.658	32.658	0.000
	Total bilirubin	4.579	0.599	0.069	7.645	0.000
	Thiols	0.325	0.065	0.139	5.009	0.000
	α-Tocopherol	5.575	1.524	0.094	3.657	0.000
	β-Carotene	29.374	28.602	0.011	1.027	0.308
	L-Ascorbic acid	0.701	0.108	0.060	6.504	0.000
5	Uric acid	2.680	0.082	0.656	32.710	0.000
	Total bilirubin	4.707	0.586	0.071	8.033	0.000
	Thiols	0.320	0.065	0.137	4.948	0.000
	α-Tocopherol	6.203	1.397	0.105	4.441	0.000
	L-Ascorbic acid	0.716	0.107	0.061	6.699	0.000

^1^ Dependent variable: TAC; weighted least square regression: weight for TAC is *w* = TAC^−2^; regression forced through the origin.

**Table 4:** Estimated values of the redox factors (extent of electronic exchange estimated at 4 min according to the CUPRAC-BCS method).

	Molecules				
	Uric Acid ^1^	Total Bilirubin	HSA-SH	α-Tocopherol	L-Ascorbic Acid
**Redox factor**	1.999	7.433	0.567	2.004	2.029
**Std. dev. ^2^**	0.009	0.084	0.008	0.030	0.013

^1^ Ref. [12]. ^2^ Std. dev. = standard deviation.

**Table 5:** Summary of the statistics for the speciation model obtained for the healthy individuals.

Summary of ANOVA Test ^1^					
	Sum of Squares	Degrees of Freedom	Variance	F	Significance
**Regression**	94,158.874	5	18,831.775	1661.090	0.000
**Prediction Error**	907.005	80	11.337		
**Total**	95,066.879	85			
**Summary of the Model**					
	**R**	**R-Square**	**Adj. R-Square**	**ROOT-MSE**	**n**
**Model**	0.995	0.990	0.990	3.367	85

^1^ Dependent variable: TAC, predictors: uric acid, total bilirubin, thiols, α-tocopherol, L-ascorbic acid. Weighted least square regression: weight for TAC is *w* = TAC^−2^, regression forced through the origin.

**Table 6:** Regression parameters optimized by multiple regression analysis for cardiopathic individuals.

Model ^1^		Unstandardized Coefficients	Standard Deviation Error	Standardized Coefficients	*t* Value	Significance
1	Uric acid	2.840	0.229	0.691	12.399	0.000
	Total bilirubin	7.527	3.549	0.118	2.121	0.050
	Thiols	0.252	0.289	0.101	0.874	0.395
	Retinol	63.479	88.673	0.084	0.716	0.484
	γ-Tocopherol	36.149	37.406	0.060	0.966	0.348
	α-Tocopherol	−6.617	18.506	−0.075	−0.358	0.725
	Lycopene	−5.841	151.063	−0.004	−0.039	0.970
	β-Carotene	92.462	292.065	0.017	0.317	0.756
	L-Ascorbic acid	3.964	3.713	0.086	1.068	0.302
2	Uric acid	2.841	0.222	0.691	12.787	0.000
	Total bilirubin	7.484	3.265	0.117	2.292	0.035
	Thiols	0.244	0.178	0.098	1.369	0.189
	Retinol	65.339	72.267	0.087	0.904	0.379
	γ-Tocopherol	36.512	35.130	0.061	1.039	0.313
	α-Tocopherol	−6.930	16.142	−0.079	−0.429	0.673
	β-Carotene	89.852	275.684	0.016	0.326	0.748
	L-Ascorbic acid	4.047	2.944	0.088	1.375	0.187
3	Uric acid	2.828	0.213	0.688	13.250	0.000
	Total bilirubin	7.484	3.183	0.117	2.352	0.030
	Thiols	0.244	0.173	0.098	1.404	0.177
	Retinol	60.784	69.121	0.081	0.879	0.391
	γ-Tocopherol	31.031	30.067	0.052	1.032	0.316
	α-Tocopherol	−4.366	13.742	−0.050	−0.318	0.754
	L-Ascorbic acid	3.987	2.864	0.087	1.392	0.181
4	Uric acid	2.806	0.197	0.682	14.279	0.000
	Total bilirubin	7.734	3.010	0.121	2.569	0.019
	Thiols	0.212	0.140	0.085	1.520	0.145
	Retinol	46.029	49.972	0.061	0.921	0.369
	γ-Tocopherol	25.719	24.393	0.043	1.054	0.305
	L-Ascorbic acid	3.467	2.294	0.076	1.511	0.147
5	Uric acid	2.784	0.194	0.677	14.326	0.000
	Total bilirubin	8.614	2.844	0.135	3.029	0.007
	Thiols	0.246	0.135	0.099	1.826	0.083
	γ-Tocopherol	40.282	18.505	0.067	2.177	0.042
	L-Ascorbic acid	4.371	2.066	0.095	2.115	0.047

^1^ dependent variable: TAC; weighted least square regression: weight for TAC is *w* = TAC^−2^; regression forced through the origin.

**Table 7:** Summary of the statistics for the speciation model obtained for the cardiopathic individuals.

Summary of ANOVA Test ^1^					
	Sum of Squares	Degrees of Freedom	Variance	F	Significance
**Regression**	15.4	5	3.08	6.416	0.000
**Prediction error**	9.60	20	0.48		
**Total**	25.00	25			
**Summary of the Model**					
	**R**	**R-Square**	**Adj. R-Square**	**ROOT-MSE**	**n**
**Model**	0.785	0.616	0.520	0.693	25

^1^ dependent variable: TAC; predictors: uric acid, total bilirubin, thiols, γ-tocopherol, L-ascorbic acid. Weighted least square regression: weight for TAC is *w* = TAC^−2^. Regression forced through the origin.

**Table 8:** Mean values of the TAC and of concentrations of the redox-active biomolecules in samples from healthy and cardiopathic individuals (males’ and females’ data mixed).

	Healthy Individuals (Mean)	Cardiopathic Individuals (Mean)	RD % ^1^
TAC (µmol L^−1^)	1002	1235	18.9
Uric acid (µmol L^−1^)	266	303	12.2
Total bilirubin (µmol L^−1^)	14	16	12.5
Thiols (µmol L^−1^)	462	368	−25.5
Retinol (nmol L^−1^)	1832	1202	−54.4
γ-Tocopherol (nmol L^−1^)	775	1272	39.1
α-Tocopherol (nmol L^−1^)	18,048	11,160	−61.7
Lycopene (nmol L^−1^)	1965	554	−254.7
β-Carotene (nmol L^−1^)	336	130	−158.5
L-Ascorbic acid (µmol L^−1^)	76	20	−280.0

^1^ RD is the relative difference percentage expressed as RD% = [(x_c_ − x_b_)/x_c_] × 100, where x_c_ is the mean value for the cardiovascular surgery patients and x_B_ is the mean value for the blood donor group.

## Data Availability

Data are contained within the article or supplementary material.

## Supplementary Materials

The following are available online at https://www.mdpi.com/article/10.3390/antiox10050656/s1, Table S1: Variables considered in the different models for healthy individuals. Table S2: Model parameters for healthy individuals. Table S3: Results of ANOVA tests for healthy individuals. Table S4 and Table S5: Results of the study of the redox reaction between Cu(II) and bilirubin at 30 min and under the CUPRAC-BCS method condition. Table S6: Contributions of plasma redox-active molecules to the TAC values of healthy individuals. Table S7: Variables considered in the different models for cardiopathic individuals. Table S8: Model parameters for cardiopathic individuals. Table S9: Results of the ANOVA test for cardiopathic individuals. Table S10: Contributions of the redox-active biomolecules to the TAC values of cardiopathic individuals.
