# Supplementary material for: Biomolecules Responsible for the Total Antioxidant Capacity (TAC) of Human Plasma in Healthy and Cardiopathic Individuals: A Chemical Speciation Model
Source: Antioxidants (Basel). 2021 Apr 23;10(5):656. doi: 10.3390/antiox10050656 (PMC8145038; doi:10.3390/antiox10050656)
Supplement: Supplementary file 1 [file antioxidants-10-00656-s001.zip › antioxidants-1176107-supplementary.pdf]

Supplementary Materials: Biomolecules Responsible for the Total Antioxidant Capacity (TAC) of Human Plasma in Healthy and Cardiopathic Individuals: A Chemical Speciation Model

Enrico Prenesti [1], Silvia Berto [1,*], Fabio Gosmaro [1,2], Marco Bagnati [3] and Giorgio Bellomo [3]

[1] Department of Chemistry, University of Turin, Via Pietro Giuria 5, 10125 Turin, Italy; enrico.prenesti@unito.it (E.P.); fabiogos@yahoo.it (F.G.)
[2] Istituto Professionale di Stato per Servizi Alberghieri e Ristorazione G. Giolitti, Piazza IV Novembre, 12080 Mondovì, Italy
[3] Clinical Chemical Analysis Laboratory of the University Hospital "Maggiore della Carità", University of Eastern Piedmont Amedeo Avogadro, C.so Giuseppe Mazzini, 18, 28100 Novara, Italy; marco.bagnati@maggioreosp.novara.it (M.B.); bellomo.giorgio@gmail.com (G.B.)
* Correspondence: silvia.berto@unito.it.

1. Significance of antioxidants as predictors of healthy individuals' TAC

Table S1 – Variables considered in the different model for healthy individuals.

Model [1]	Variables in	Variables out	Procedures
**1**	Uric Acid, Total Bilirubin, Thiols, Retinol, γ-Tocopherol, α-Tocopherol, Lycopene, β-Carotene, Ascorbic acid	---	---
**2**	Uric Acid, Total Bilirubin, Thiols, Retinol, γ-Tocopherol, α-Tocopherol, β-Carotene, Ascorbic acid	Lycopene	Backward removal (removal criterion: $p \geq 0.10$)
**3**	Uric Acid, Total Bilirubin, Thiols, Retinol, α-Tocopherol, Lycopene, β-Carotene, Ascorbic acid	γ-Tocopherol	Backward removal (removal criterion: $p \geq 0.\ 10$)
**4**	Uric Acid, Total Bilirubin, Thiols, γ-Tocopherol, α-Tocopherol, Lycopene, β-Carotene, Ascorbic acid	Retinol	Backward removal (removal criterion: $p \geq 0.\ 10$)
**5**	Uric Acid, Total Bilirubin, Thiols, Retinol, γ-Tocopherol, α-Tocopherol, Lycopene, Ascorbic acid	β-Carotene	Backward removal (removal criterion: $p \geq 0.\ 10$)

[1] dependent variable: TAC; weighted least square regression: weight for TAC is $w = TAC^{-2}$; regression forced through the origin.

Table S2 – Model parameters for healthy individuals.

Model [1]	R	R-square	Adj. R-square	ROOT-MSE
**1**	0.999	0.998	0.998	1.49651
**2**	0.999	0.998	0.998	1.48846
**3**	0.999	0.998	0.998	1.48130

**4**	0.999	0.998	0.998	1.47967
**5**	0.999	0.998	0.998	1.48018

[1] dependent variable: TAC; weighted least square regression: weight for TAC is w = TAC^{-2}; regression forced through the origin,
model 1: Predictors: urate, total bilirubin, thiols groups, retinol, γ-tocopherol, α-tocopherol, lycopene, β-carotene, L-ascorbic acid
model 2 - Predictors: urate, total bilirubin, thiols groups, retinol, γ-tocopherol, α-tocopherol, β-carotene, L-ascorbic acid
model 3 - Predictors: urate, total bilirubin, thiols groups, retinol, α-tocopherol, β-carotene, L-ascorbic acid
model 4 - Predictors: urate, total bilirubin, thiols groups, α-tocopherol, β-carotene, L-ascorbic acid
model 5 - Predictors: urate, total bilirubin, thiols groups, α-tocopherol, L-ascorbic acid.

Table S3 – Results of ANOVA test for healthy individuals.

**Model** [1]		**Sum of square**	**Degree of freedom**	**Variance**	**F**	**Significance**
	Regression	93040.936	9	10337.882	4616.095	0.000
**1**	Prediction error	170.204	76	2.240		
	Total	93211.140	85			
	Regression	93040.545	8	11630.068	5249.376	0.000
**2**	Prediction error	170.595	77	2.216		
	Total	93211.140	85			
	Regression	93039.989	7	13291.427	6057.399	0.000
**3**	Prediction error	171.151	78	2.194		
	Total	93211.140	85			
	Regression	93038.175	6	15506.362	7082.358	0.000
**4**	Prediction error	172.965	79	2.189		
	Total	93211.140	85			
	Regression	93035.865	5	18607.173	8492.807	0.000
**5**	Prediction error	175.275	80	2.191		
	Total	93211.140	85			

[1] dependent variable: TAC; weighted least square regression: weight for TAC is w = TAC^{-2}; regression forced through the origin,
model 1: Predictors: urate, total bilirubin, thiols groups, retinol, γ-tocopherol, α-tocopherol, lycopene, β-carotene, L-ascorbic acid
model 2 - Predictors: urate, total bilirubin, thiols groups, retinol, γ-tocopherol, α-tocopherol, β-carotene, L-ascorbic acid
model 3 - Predictors: urate, total bilirubin, thiols groups, retinol, α-tocopherol, β-carotene, L-ascorbic acid
model 4 - Predictors: urate, total bilirubin, thiols groups, α-tocopherol, β-carotene, L-ascorbic acid
model 5 - Predictors: urate, total bilirubin, thiols groups, α-tocopherol, L-ascorbic acid.

2. Redox factors

Study of the redox reaction between Cu(II) and Bilirubin

Six separate series of bilirubin standard solutions (concentrations: 2.5, 5.0, 12.5, 25, 50 and 100 μmol L^{-1}) were prepared and analyzed in three replicates. The reaction under study was left up to the signal saturation (time reaction = 30 min) to estimate the electronic exchange between Cu(I) and bilirubin. Results reported in Table 4S show experimental slope equal to 8.044 with standard deviation of 0.223, indicating an electronic exchange equal to eight for the redox reaction under consideration.

Table S4 – Results of the study of the redox reaction between Cu(II) and Bilirubin at 30 min.

Summary of the coefficients [1]					
	**Value**	**Standard deviation**	**t-value**	**Significance**	
**Slope**	8.044	0.223	36.135	0.000	
**Intercept**	3.119	0.047	66.075	0.000	
**Summary of ANOVA test**					
	**Sum of squares**	**Degrees of freedom**	**Variance**	**F**	**Significance**
**Model**	625.210	1	625.210	1305.753	0.000
**Prediction error**	7.661	16	0.479		
**Total**	632.871	17			
**Summary of the model**					
	**R**	**R-square**	**Adj. R-square**	**ROOT-MSE**	**N**
**Model**	0.994	0.988	0.987	0.692	18

[1] predictor: $C_{Bilirubin}$, dependent variable: $C_{Cu(I)}$, weighted least square regression: weight for $C_{Bilirubin}$ is $w = C_{Bilirubin}^{-1.5}$.

Since the aim of the study is focused on the investigation of redox reaction between Cu(II) and bilirubin under the CUPRAC-BCS method condition (time reaction = 4 min), the amount of the electronic exchange at 4 min was estimated.

Results in Table 5S show experimental slope equal to 7.433 with standard deviation of 0.084.

Table S5 – Results of the study of the redox reaction between Cu(II) and Bilirubin under the CUPRAC-BCS method condition.

Summary of the coefficients [1]					
	**Value**	**Standard deviation**	**t-value**	**Significance**	
**Slope**	7.433	0.084	88.997	0.000	
**Intercept**	2.350	0.059	40.165	0.000	
**Summary of ANOVA test**					
	**Sum of squares**	**Degrees of freedom**	**Variance**	**F**	**Significance**
**Model**	651.848	1	651.848	7920.524	0.000
**Prediction error**	1.371	16	0.082		
**Total**	653.165	17			
**Summary of the model**					
	**R**	**R-square**	**Adj. R-square**	**ROOT-MSE**	**N**
**Model**	0.999	0.998	0.998	0.287	18

[1] predictor: $C_{Bilirubin}$, dependent variable: $C_{Cu(I)}$, weighted least square regression: weight for $C_{Bilirubin}$ is $w = C_{Bilirubin}^{-0.5}$.

3. Speciation model of the TAC

Table S6 – Contributions of plasma redox active molecules to the TAC of healthy individuals.

Substance	n	Redox Factor	Min (%)	Max (%)	Mean ± SE [1] (%)
Uric Acid	85	2.0	32.50	62.69	47.97 ± 0.65
Total Bilirubin	85	7.4	3.25	44.24	9.50 ± 0.61
Thiols	85	0.6	15.29	37.20	25.83 ± 0.52
α-Tocopherol	85	2.0	1.98	5.45	3.36 ± 0.09
L-Ascorbic acid	85	2.0	2.07	55.51	14.63 ± 1.15
TOTAL	85				101.29 ± 3.02

[1] SE is the symbol for standard deviation error. SE is expressed as SD/√n where SD is the standard deviation.

4. Significance of antioxidants as predictors of cardiopathic individuals' TAC

Table S7 – Variables considered in the different model for cardiopathic individuals.

Model [1]	Variables in	Variables out	Procedures
**1**	Uric Acid, Total Bilirubin, Thiols, Retinol, γ-Tocopherol, α-Tocopherol, Lycopene, β-Carotene, L-Ascorbic acid	---	---
**2**	Uric Acid, Total Bilirubin, Thiols, Retinol, γ-Tocopherol, α-Tocopherol, β-Carotene, L-Ascorbic acid	Lycopene	Backward removal (removal criterion: $p \geq 0.10$)
**3**	Uric Acid, Total Bilirubin, Thiols, Retinol, γ-Tocopherol, α-Tocopherol, Lycopene, L-Ascorbic acid	β-Carotene	Backward removal (removal criterion: $p \geq 0.\ 10$)
**4**	Uric Acid, Total Bilirubin, Thiols, Retinol, γ-Tocopherol, Lycopene, β-Carotene, L-Ascorbic acid	α-Tocopherol	Backward removal (removal criterion: $p \geq 0.\ 10$)
**5**	Uric Acid, Total Bilirubin, Thiols, γ-Tocopherol, α-Tocopherol, Lycopene, β-Carotene, L-Ascorbic acid	Retinol	Backward removal (removal criterion: $p \geq 0.\ 10$)

[1] dependent variable: TAC; weighted least square regression: weight for TAC is $w = TAC^{-2}$; regression forced through the origin.

Table S8 – Model parameters for cardiopathic individuals.

Model [1]	R	R-square	Adj. R-square	ROOT-MSE
**1**	0.995	0.989	0.983	0.129
**2**	0.995	0.989	0.984	0.125
**3**	0.995	0.989	0.985	0.122
**4**	0.995	0.989	0.986	0.119

5	0.994	0.989	0.986	0.119

[1] dependent variable: TAC; weighted least square regression: weight for is w = TAC^{-2}; regression forced through the origin,
model 1 - Predictors: Uric Acid, Total Bilirubin, Thiols, Retinol, γ-Tocopherol, α-Tocopherol, Lycopene, β-Carotene, L-Ascorbic acid;
model 2 - Predictors: Uric Acid, Total Bilirubin, Thiols, Retinol, γ-Tocopherol, α-Tocopherol, β-Carotene, L-Ascorbic acid;
model 3 - Predictors: Uric Acid, Total Bilirubin, Thiols, Retinol, γ-Tocopherol, α-Tocopherol, L-Ascorbic acid;
model 4 - Predictors: Uric Acid, Total Bilirubin, Thiols, Retinol, γ-Tocopherol, L-Ascorbic acid;
model 5 - Predictors: Uric Acid, Total Bilirubin, Thiols, γ-Tocopherol, L-Ascorbic acid.

Table S9 – Results of the ANOVA test for cardiopathic individuals.

Model [1]		Sum of square	Degree of freedom	Variance	F	Significance
	Regression	24.732	9	2.748	164.279	0.000
**1**	Prediction error	0.268	16	0.017		
	Total	25.000	25			
	Regression	24.732	8	3.092	196.346	0.000
**2**	Prediction error	0.268	17	0.016		
	Total	25.000	25			
	Regression	24.731	7	3.533	236.104	0.000
**3**	Prediction error	0.269	18	0.015		
	Total	25.000	25			
	Regression	24.729	6	4.122	289.119	0.000
**4**	Prediction error	0.271	19	0.014		
	Total	25.000	25			
	Regression	24.717	5	4.943	349.421	0.000
**5**	Prediction error	0.283	20	0.014		
	Total	25.000	25			

[1] dependent variable: TAC; weighted least square regression: weight for is w = $TAC^{-2;}$ regression forced through the origin;
model 1 - Predictors: Uric Acid, Total Bilirubin, Thiols, Retinol, γ-Tocopherol, α-Tocopherol, Lycopene, β-Carotene, L-Ascorbic acid;
model 2 - Predictors: Uric Acid, Total Bilirubin, Thiols, Retinol, γ-Tocopherol, α-Tocopherol, β-Carotene, L-Ascorbic acid;
model 3 - Predictors: Uric Acid, Total Bilirubin, Thiols, Retinol, γ-Tocopherol, α-Tocopherol, L-Ascorbic acid;
model 4 - Predictors: Uric Acid, Total Bilirubin, Thiols, Retinol, γ-Tocopherol, L-Ascorbic acid;
model 5 - Predictors: Uric Acid, Total Bilirubin, Thiols, γ-Tocopherol, L-Ascorbic acid.

Table S10 – Contributions of the redox active biomolecules to the TAC of cardiopathic individuals.

Substance	n	Redox Factor	Min (%)	Max (%)	Mean ± SE [1] (%)
Urate	25	2.0	30.78	64.86	47.61 ± 2.05
Bilirubin	25	7.4	3.22	27.80	9.95 ± 1.22
Thiols	25	0.6	8.09	48.97	21.26 ± 2.31
γ-Tocopherol	25	2.0	0.01	1.19	0.22 ± 0.05

L-Ascorbic acid	25	2.0	1.01	11.77	3.65 ± 0.48
Total	25				82.69 ± 6.11

[1] SE is the symbol for standard deviation error. SE is expressed as SD/√n where SD is the standard deviation.
